# Meta-research on key metrics of preregistered scoping reviews

**DOI:** 10.1017/rsm.2026.10078

**Published:** 2026-06-15

**Authors:** Kim Nordmann, Paula Kuhn, Florian Fischer

**Affiliations:** Bavarian Research Center for Digital Health and Social Care, Kempten University of Applied Sciences, Kempten, Germany

**Keywords:** evidence synthesis, methodological metrics, protocol registration, publication trends, research methodology, scoping review

## Abstract

Scoping reviews are increasingly used for evidence synthesis, yet a comprehensive overview of benchmarks for their conduct is lacking. This study aimed to extract and analyze key metrics of preregistered scoping reviews to aid researchers in planning, resource allocation, methodological rigor, and innovation. We examined 2,038 scoping review protocol registrations on the Open Science Framework between April and August 2024, identifying 891 corresponding publications. Extracted variables included review type (e.g., rapid scoping review), information about the number of studies in the screening process, methodological practices, and funding. Using Stata, we calculated process metrics such as review duration, yield rate, and team composition. Qualitative analysis was applied to further metrics, including aims and eligibility criteria frameworks. Among the 891 publications, 91.47% were published as scoping reviews. On average, reviews involved 6.11 authors and took 80.41 weeks from registration to publication, with rapid scoping reviews completed in 55.63 weeks. Most publications (80.13%) originated from high-income countries, where funding was more common. An average of 5.19 databases were searched, identifying a mean of 6,920.66 potential inclusions, with 92.64 studies ultimately included (yield rate: 5.68%). Endnote was the most frequently employed screening tool. Language restrictions were reported in 37.60% of cases, and 75.42% did not conduct or report critical appraisal. This study provides valuable insights into common practices within scoping review processes, offering researchers a foundation to allocate resources more efficiently and enhance research quality through methodological benchmarks. It underscores the need for greater transparency and improved methodological rigor.

## Highlights

### What is already known?

- Scoping reviews are an evidence synthesis method widely used to map existing literature and identify research gaps. Compared to systematic reviews, they generally encompass a broader scope of literature.
- While the Preferred Reporting Items for Systematic Reviews and Meta-Analyses-ScR guidelines provide a framework for reporting on scoping reviews, comprehensive data on key metrics specific to scoping reviews—such as duration and yield rate—remain limited.

### What is new?

- We systematically analyzed key metrics of 2,038 scoping review protocol registrations on the Open Science Framework and 891 corresponding publications. Key findings include the average time from registration to publication, study inclusion rates, and common methodological practices.

### Potential impact for *RSM* readers

- Evaluating these metrics informs researchers, reviewers, and other stakeholders. The findings can aid in resource planning, improve methodological rigor, and enhance transparency in scoping review processes.

## 1 Introduction

Scoping reviews are a distinct and novel methodology for evidence synthesis, initially described in the early 2000s.^1^ In contrast to systematic reviews, which aim to answer a particular question and assess the risk of bias in primary studies and in evidence syntheses overall, scoping reviews are used to map the evidence in a field and identify research gaps.^1^^,^^2^ This provides space for exploring and evaluating concepts that are difficult to investigate in systematic reviews.^1^^,^^3^ Consequently, the research questions in scoping reviews are broader, and scoping reviews potentially include more heterogeneous studies with a wider scope.^4^ Since their introduction, scoping reviews have been increasingly used, with PubMed recording over 12,000 hits for scoping reviews in 2024 alone.^5^^,^^6^

This growing adoption has been accompanied by a rapid evolution and maturation of scoping review methodology, first outlined in 2005.^1^^,^^7^^,^^8^ Since 2018, scoping reviews have been supported by thorough methodological guidance: The Preferred Reporting Items for Systematic Reviews and Meta-Analyses (PRISMA) guidelines provide a structured and extensive framework for authors reporting on systematic reviews and meta-analyses. Based on this foundation, the PRISMA Extension for Scoping Reviews (PRISMA-ScR) expanded the framework by including 22 reporting items and effective recommendations for reporting on scoping reviews.^9^ The 2020 update of the Manual for Evidence Synthesis by the Joanna Briggs Institute (JBI) further strengthened the scoping review methodology with a dedicated chapter.^10^

Despite the well-founded methodology, an overview of key metrics of scoping reviews, such as time to publication, average yield rate, and common use cases, does not yet exist. While studies have elicited these for systematic reviews, they cannot be extrapolated to scoping reviews due to the differences in scope and methodology.^11^^,^^12^ Such key metrics, however, are necessary to inform researchers with regard to planning and resource allocation, to manage the expectations of stakeholders, and to enhance methodological rigor by understanding common practices and outcomes, which, in turn, create opportunities to develop innovative research questions and generate new ideas.^13^^,^^14^ Therefore, this study aimed to elicit key metrics of preregistered scoping reviews, including team composition, the time from registration to publication, type and field of the review, structure and nature of eligibility criteria, primary resources, search conduct and efficiency, critical appraisal, and funding. Specifically, we aim to respond to the following research questions:

1. What are common methodological characteristics of preregistered scoping reviews?
2. What challenges or gaps exist in the current conduct of scoping reviews, and how might these be addressed through clearer methodological guidance?

## 2 Methodology

We structured our study following the methodology used by Borah et al., who relied on PROSPERO to identify preregistered systematic reviews, retrieved the corresponding peer-reviewed articles of their results, and analyzed their key metrics.^12^

### 2.1 Study selection

As for systematic reviews, it is highly recommended to preregister scoping reviews to minimize duplication of efforts, reduce bias, elicit feedback from the research community, and improve manuscripts.^10^^,^^15^^,^^16^ However, PROSPERO does not support registering scoping reviews, and due to a lack of a common registry, many opt to either publish a scoping review protocol paper in a scientific journal or use open-access databases such as the Open Science Framework (OSF), figshare, or—if working with the JBI—the JBI Systematic Review Register.^11^^,^^16^ OSF allows uploading projects, preprints, and files to register projects, such as scoping reviews, and is not limited to a certain research sector.^15^ As such, we focused our study on scoping review protocols registered in OSF and their corresponding publication.

**Figure 1:**
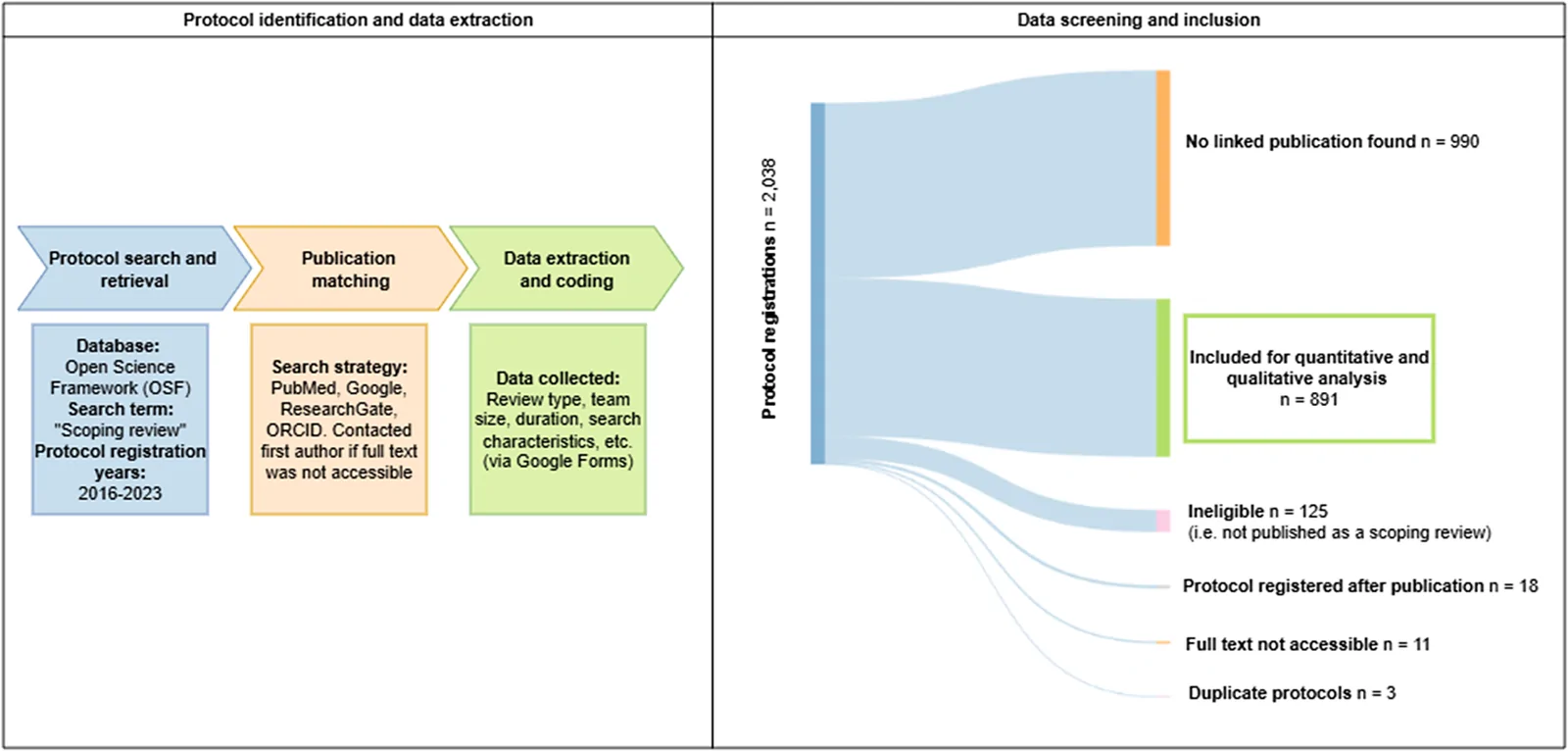
Process of protocol registration identification, data extraction, data screening, and inclusion. Figure 1 long description.

During the retrieval phase between March and August 2024, we identified registered scoping review protocols via the tab “Registrations” in the OSF using the search term “Scoping review” (see Figure 1). In registrations, researchers can upload outlines of original research studies, any adjustments made during the process, and the project results.^15^ We identified 2,038 scoping review registrations on the OSF by fully assessing all entries identified according to the above methodology for 2016–2020. Despite OSF being launched in 2013, we selected 2016 as the starting point due to low registration numbers until then and the increasing popularity of the open science movement in recent years.^17^

Due to the large yield in 2021–2023, we analyzed the first ten percent of all registrations when ranked by relevance, identifying 221 registrations out of 2,206 entries analyzed in 2021; 313 out of 3,130 in 2022; and 415 out of 4,150 in 2023. Registrations were included if they met the criteria outlined in Table 1. If a registration was updated, we extracted the most recent data provided in OSF. An official update on OSF facilitated this process from May 2023 onward, allowing users to switch between the original registration and the updated versions, with clear visibility of changes, timestamps, and reasons for updates.^18^ Prior to this, registrations were updated in a new entry, resulting in a manual update of the previous entry in our database.

**Table 1:** Inclusion and exclusion criteria for scoping review registrations Table 1 long description.

Inclusion criteria	Exclusion criteria
All types of scoping reviews (systematic scoping review, rapid scoping review)	Any other registration type
Full registrations	Incomplete registrations, registration components
	Registration withdrawn
	Publication of the results within four weeks of registering the protocol
	Registration of the protocol after publication of results

To retrieve published results of these 2,038 scoping review registrations, we searched databases, that is, PubMed, for peer-reviewed articles using the registration title. We considered it a match if at least one author coincided. If unsuccessful, we used Google, ResearchGate, and ORCID using the protocol registration title and the first author’s name in the registration. If that was equally unsuccessful, we searched them for the last author. In cases where the publications were unavailable through open access or institutional access, we contacted the authors via email to obtain access to their publications. Registrations without published results (990 registrations) or published under another review type, such as systematic review (125 registrations), those registered after publication (18 registrations), and duplicates (3 registrations), were not included in the analysis. Registrations without access to the publication after contacting the authors (11 registrations) were also excluded.

### 2.2 Data extraction

In total, data from 891 publications were extracted by PK using a Google form, subject to random verifications by KN (see Appendix 1 of the Supplementary Material for a full list of citations and Appendix 2 of the Supplementary Material for the Google form). We used the Google form to extract the following information: type of scoping review; dates of registration and publication; number of team members and authors; country of the first author’s institution; abstract; aim; type, number, and names of databases searched; eligible sources of primary evidence included in the analysis (i.e., articles, book chapters, and gray literature); inclusion and exclusion criteria along with the search strategy; tool(s) used for screening; the number of studies in each stage provided in the PRISMA diagram and/or the text; the interrater agreement; a link to all studies included in the publication; and funding information.

#### 2.2.1 Team composition

We evaluated the number of team members listed in the OSF registration, the number of authors of the publication, and the overlap between authors and team members between the OSF protocol registration and the publication of results. If individual authors were not listed in publications, for example, by mentioning a research group, we excluded the publication from this section of our analysis (*n* = 4 registrations). However, these publications were still included elsewhere. We further identified the country of the first author’s primary institution in the publication and categorized these countries into high-, upper-middle-, lower-middle-, and low-income based on the World Bank’s Gross National Income (GNI).^19^

#### 2.2.2 Timeline

We extracted the dates of protocol registration and publication and calculated the duration of the scoping review project using the time difference between the registration and online publication dates. In cases where the publication date was unavailable, we used the acceptance date for our analysis. Furthermore, we calculated the proportion of registered protocols that had been published by the time of our analysis, along with the average time to publication, stratified by the year in which each protocol was published.

#### 2.2.3 Search conduct and efficiency

We recorded the eligible sources of primary evidence as stated in the PRISMA diagram in the published article, as well as the type, number, and names of databases searched. In cases where only the database provider was mentioned, we extracted this information and identified the database afterward. We categorized the databases into thematic groups based on the primary focus: medical and health (i.e., PubMed, Embase); engineering and technology (i.e., IEEE Xplore, Compendex); social sciences and humanities (i.e., SocINDEX); multidisciplinary (i.e., SciELO Data, Wiley Online Library); government and policy-focused (i.e., PAIS Index); other specialized databases (i.e., AGRICOLA, EconLit); systematic review databases (i.e., Cochrane Library, PROSPERO); open access repositories (i.e., directory of open access journals); thesis and dissertation databases (i.e., EThOS); and gray literature sources and websites (i.e., the US Centers for Disease Control and Prevention website) (see Appendix 3 of the Supplementary Material for a comprehensive list). We also extracted information about the number of publications at each step of selecting the literature according to our extraction categories from the PRISMA diagram provided in the publication, or—if missing—from the supplementary material. Publications identified outside of database searches, through sources such as websites or gray literature searches, were combined with those retrieved from the database to determine the total number of studies retrieved in the literature search.^12^ Finally, we extracted digital tools facilitating the screening process, such as EndNote or Covidence, and, if provided, the interrater agreement during abstract and full-text screening.

#### 2.2.4 Scope of the review and critical appraisal

We recorded the type of scoping review (scoping review, rapid scoping review, and systematic scoping review) in both the registration and the published article. All three types of scoping review aim to explore the extent, range, and nature of existing evidence.^3^^,^^4^ While the distinction between “scoping review” and “systematic scoping review” remains poorly defined, a systematic scoping review typically suggests the integration of systematic review methods and features. Rapid reviews, in turn, prioritize efficiency and speed, often through a narrow focus, streamlined methods, or restricted searches and research questions.^20^^,^^21^ We further recorded the language of the review, abstract, research questions, and explicitly stated aims, inclusion and exclusion criteria, the search strategy, and the conduct and nature of critical appraisal.

#### 2.2.5 Funding

Information about funding was extracted from the registration and the published article. We recorded whether funding was received and, if so, the source of the funding. Additionally, we separately documented funding received by individual authors, such as salary support.

### 2.3 Data analysis

The extracted data in the Google Forms was downloaded and cleaned in Microsoft Excel (Microsoft Corporation, Version 16). Analysis was performed using Stata (StataCorp LLC, Version 18) for quantitative analysis. The yield rate was calculated according to equation (1), based on the numbers reported in the PRISMA diagram. Throughout this analysis, we refer to this metric as search efficiency to highlight its role in evaluating the effectiveness of the search and screening process:

(1) $$\begin{array}{r} Yield\;\;rate=\frac{\#Articles\;\;included}{\#Articles\;found\;in\;literature\;search-Duplicates} \end{array}$$

We performed inductive qualitative analysis on the reviews’ aims, frameworks applied for eligibility criteria, and limitations of inclusion criteria, article fields according to Medical Subject Headings (MeSH) terms, and funding sources. The aims were classified into one or more of the inductively derived categories: assess interventions, develop frameworks or models, identify factors or barriers, identify gaps, map evidence, or understand outcomes and impacts. For the eligibility criteria, we analyzed the use of frameworks, such as Population, Concept, Context (PCC), frequently used to answer broad questions without interventions, or Population, Intervention, Comparison, Outcome (PICO), which is predominantly used in clinical research.^22^^,^^23^ We also assessed language and time-frame restrictions. Using the MeSH terms provided in PubMed, or the journal’s subject matter if MeSH terms were not provided, we inductively established six thematic groups to classify the article fields: clinical and patient-centered care, healthcare systems and innovation, public health and population health, rehabilitation and wellness, research and methodology, and specialized fields. The predominance of categories in health and healthcare stemmed from the articles available for our analysis rather than a deliberate selection. Funding sources were classified into foundations and charities, governmental or state-sponsored funding, research and medical organizations, private companies and corporations, universities and academic institutions, university research fellowships and grants, and multisource funding.

We conducted a correlation analysis to examine the relationship between various key metrics, including the relation (1) between funding and, for example, country, critical appraisal, and thematic field; (2) between metrics, for example, number of authors, aim, number of included publications, and use of frameworks on the time from registration to publication; and (3) between, for example, number of authors, review type, and screening tools on the presence of critical appraisal. Additionally, we explored the impact of review aims, frameworks, thematic fields, and language on outcomes and characteristics such as publication timelines, database use, and the yield rate.

## 3 Results

### 3.1 Type of scoping review and field

Of the 891 scoping review protocol registrations on OSF with corresponding publications, 845 (94.84%) were standard scoping reviews, 37 (4.15%) were labeled as systematic scoping reviews, and 9 (1.01%) as rapid scoping reviews. Among those registrations, 815 (91.47%) were ultimately published as scoping reviews, 62 (6.96%) as systematic scoping reviews, 7 (0.79%) as rapid reviews, and 7 (0.79%) were labeled differently, such as scoping reviews with a Delphi study, z-curve analysis, and meta-analysis. For the 845 standard scoping review registrations, 799 (94.56%) were published as scoping reviews, while 38 (4.50%) were published as systematic reviews, 7 (0.83%) as other types of reviews, and 1 (0.12%) as a rapid review. Among the 37 systematic scoping review registrations, 24 (64.86%) were published as systematic scoping reviews, while 13 (35.14%) were published as scoping reviews. Of the nine rapid scoping reviews registered, six (66.67%) were published as rapid scoping reviews and three (33.33%) as scoping reviews.

**Figure 2:**
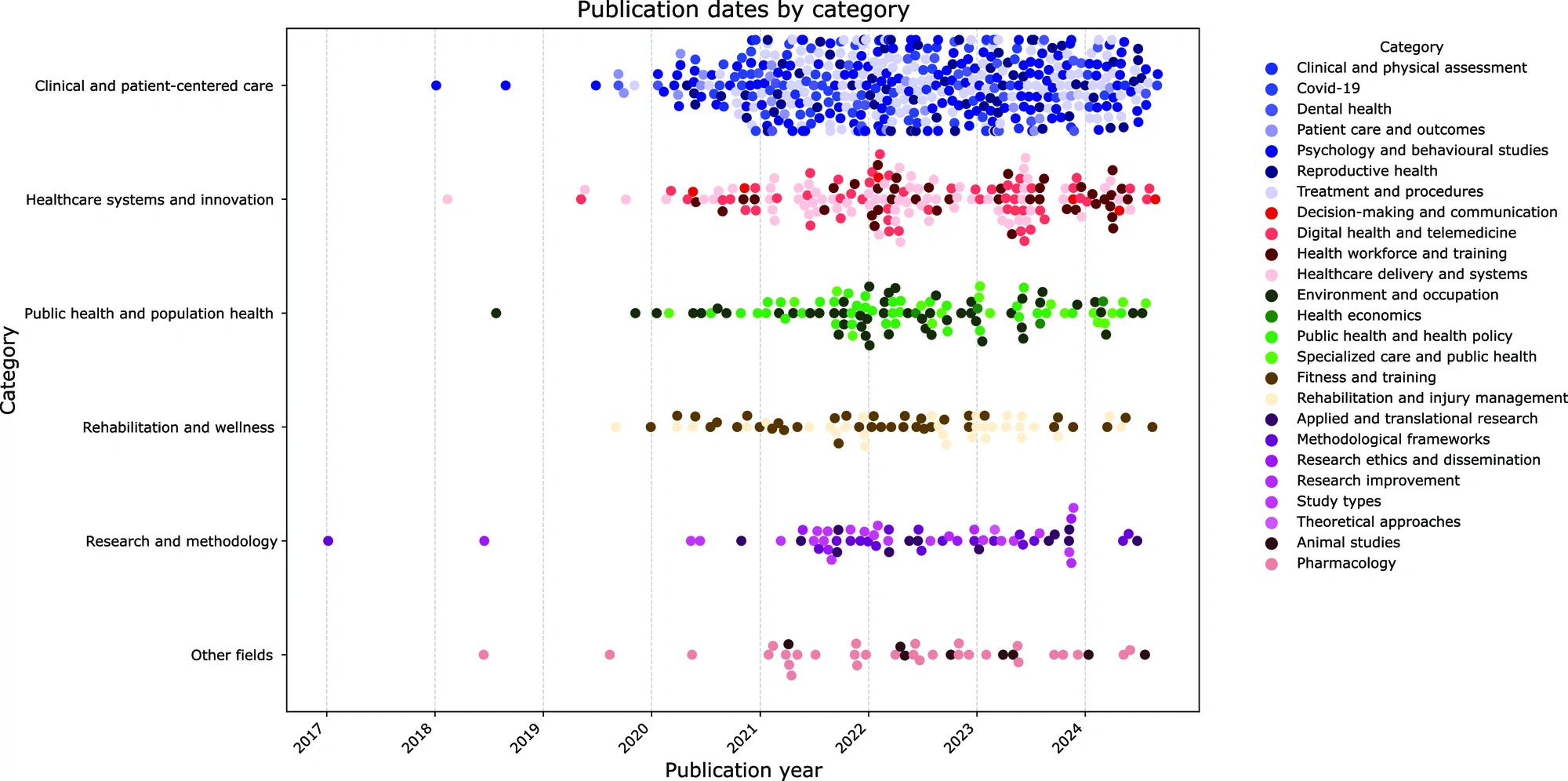
Thematic classification of publications (n = 891) and corresponding subgroups by date. Each dot represents one publication over the year of publication. Figure 2 long description.

The number of scoping review registrations rose from five in 2016 to 4,150 in 2023. All scoping reviews (*n* = 5) registered in 2016 were published by the time of our analysis (see Table 2). Scoping reviews predominantly focused on clinical and patient-centered care (*n* = 458, 51.40%), including dental and reproductive health. Some 167 (18.74%) scoping reviews revolved around healthcare systems and innovation, covering fields such as digital health and telemedicine, healthcare and delivery systems, and 107 (12.01%) scoping reviews reported upon public health and population health, including health economics and health policy. A smaller number of scoping reviews covered rehabilitation and wellness (*n* = 66, 7.41%); research and methodology (*n* = 58, 6.51%); and other fields, including animal studies and pharmacology (*n* = 35, 3.93%). The field of scoping reviews was neither associated with the number of databases searched nor the first author’s country. However, across all thematic fields, publication activity increased over time, peaking for most fields between 2021 and 2023 (see Figure 2). Scoping reviews on public and population health experienced a sharp rise between 2017 and 2023, increasing from 0% to 50.47%.

**Table 2:** Summary of articles registered and published Table 2 long description.

Year of registration	Total number of registrations	Total number of published articles by time of analysis	% of articles published by time of analysis	Average weeks to publish
2016	5	5	100	N/A
2017	5	4	80	20.14
2018	43	28	65.12	54.36
2019	190	106	55.79	63.10
2020	846	490	57.92	46.74
2021	221	103	46.61	61.75
2022	313	104	33.11	89.68
2023	415	51	12.29	99.76

The primary objective of 399 (44.78%) publications was to map evidence. Other objectives included assessing interventions (*n* = 253, 28.40%), identifying research gaps (*n* = 111, 12.46%), understanding outcomes and impacts (*n* = 87, 9.76%), developing frameworks or models (*n* = 66, 7.41%), and identifying barriers and facilitators (*n* = 21, 2.36%) (see Table 3). Some 4.60% of publications explicitly mentioned multiple objectives. No significant correlations were observed between different study aims, time to publication, critical appraisal, eligible sources of primary evidence, or the use of eligibility frameworks for study selection.

**Table 3:** Summary of the key metrics Table 3 long description.

	Total number of publications	% of publications
	891	100.00
*Type of scoping review published*		
Scoping review	815	91.47
Systematic scoping review	62	6.96
Rapid scoping review	7	0.79
Others	7	0.79
*Country GNI classification^a^*		
High	714	80.13
Upper-middle	151	16.95
Lower-middle	23	2.58
Low	3	0.34
Databases*		
Medicine and health	857	96.18
Multidisciplinary	516	57.91
Engineering and technology	377	42.31
Systematic reviews databases	295	33.11
Social sciences and humanities	67	7.52
Open access repositories	37	4.15
Government and policy	9	1.01
Gray literature and websites	8	0.90
Theses and dissertations	7	0.79
Other specialized databases	125	14.03
Aims*		
Map existing evidence	399	44.78
Assess interventions	253	28.40
Identify gaps	111	12.46
Understand outcomes and impact	87	9.76
Develop new frameworks and models	66	7.41
Identify barriers and facilitators	21	2.36
*Eligibility criteria*		
PCC	141	15.82
PICO	31	3.48
PICOS	22	2.47
Other frameworks	10	1.12
No framework reported	687	77.10
Eligible primary sources of evidence*		
Research articles	881	98.88
Gray literature	313	35.13
Abstracts	43	4.83
Books and chapters	22	2.47
Letters and commentaries	15	1.68
Editorials	10	1.12
Organizational reports	6	0.67
Other sources	15	1.68
*Critical appraisal*		
Yes	219	24.58
No	243	27.27
Not available	429	48.15
*Screening tools*		
One or more screening tools	684	76.77
None or none reported	207	23.23
*Funding*		
Yes	492	55.22
No funding received	262	29.41
No funding reported	137	15.38

*Note:* *This variable allowed for multiple responses.

a Classification based on GNI.

### 3.2 Timeline

On average, scoping reviews took 81.22 weeks (standard deviation [SD] = 43.82) from registration to publication, with completion times ranging from 5.71 to 238.29 weeks. Rapid scoping reviews were published 31.33% faster (55.63 weeks, *n* = 7; SD = 36.83 weeks) than scoping and systematic scoping reviews (81.01 weeks, *n* = 877; SD = 44.76 weeks). The number of published articles relative to registrations included in our study significantly declined over time, falling to 33.11% of registered protocols from 2022 published by August 2024 (see Table 2).

### 3.3 Team composition

**Figure 3:**
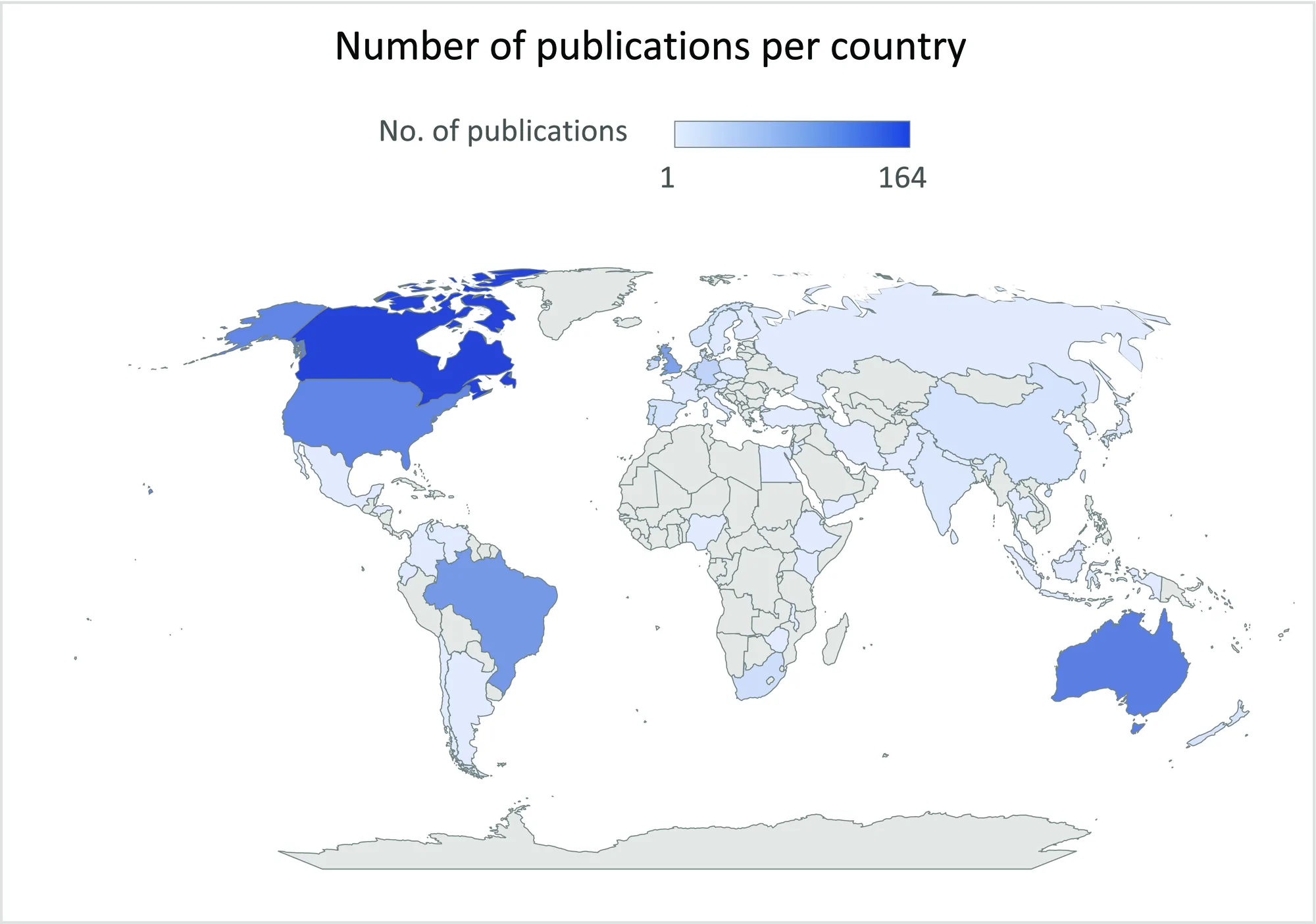
World map showing the distribution of publications by country. Darker blue shades indicate a higher number of articles published. Figure 3 long description.

On average, 3.37 (SD = 3.12, range 1–39) team members were listed in registrations, while 6.11 (SD = 3.57, range: 2–38) authored the publication. The overlap between registration and publication authorship was 3.20 authors (SD = 2.86, range 1–37). In most cases (*n* = 636, 71.38%), publications included more authors than registrations, with an average increase of 2.74 authors per article (SD = 3.21). Conversely, 3.48% of registrations (*n* = 31) had fewer authors in the publication, with an average reduction of 1.53 authors per publication (SD = 1.19). Geographically, 80.13% of publications originated from high-income countries (*n* = 714), 16.95% from upper-middle-income countries (*n* = 151), 2.58% from lower-middle-income countries (*n* = 23), and 0.34% from low-income countries (*n* = 3). In total, publications spanned 58 countries, with the most frequent first-author affiliations in Canada (18.41%, *n* = 164); Australia (13.36%, *n* = 119); and the United States (12.57%, *n* = 112) (see Figure 3). In contrast, 17 countries contributed only a single article.

Publications with more authors tended to take longer to publish (*r* = 0.16): Publications in the top 10% longest duration had an average of 7.76 authors (SD 6.10, range: 2–38), whereas those in the shortest 10% duration listed an average of 5.58 authors (SD 2.74, range: 2–17).

### 3.4 Search conduct and efficiency

The analysis of the number and type of eligible sources of primary evidence used for inclusion revealed that 566 publications (63.52%) relied on a single source. Two eligible sources of primary evidence were used in 277 publications, and three eligible sources of primary evidence were used in 35 (3.93%) publications. Some six (2.92%) included more than three eligible sources of primary evidence. Research articles were the most included eligible sources of primary evidence (*n* = 881, 98.88%), followed by gray literature (*n* = 313, 35.13%). Other sources, such as abstracts, books, and editorials, were included less frequently (see Table 3).

A search strategy for identifying relevant studies was reported in 90.80% (*n* = 809) of publications, either within the article or the supplementary data. However, there was great variation in the quality and detail of the reported search strategies. On average, 5.21 databases were searched (SD = 2.47, range: 0–20). Ten publications (1.12%) did not screen databases but searched websites or app stores exclusively. In total, 293 distinct databases were used, with medical and health-related databases being most frequently utilized (96.18% of publications), followed by multidisciplinary databases (57.91%) and those focusing on engineering and technology (42.31%).

**Figure 4:**
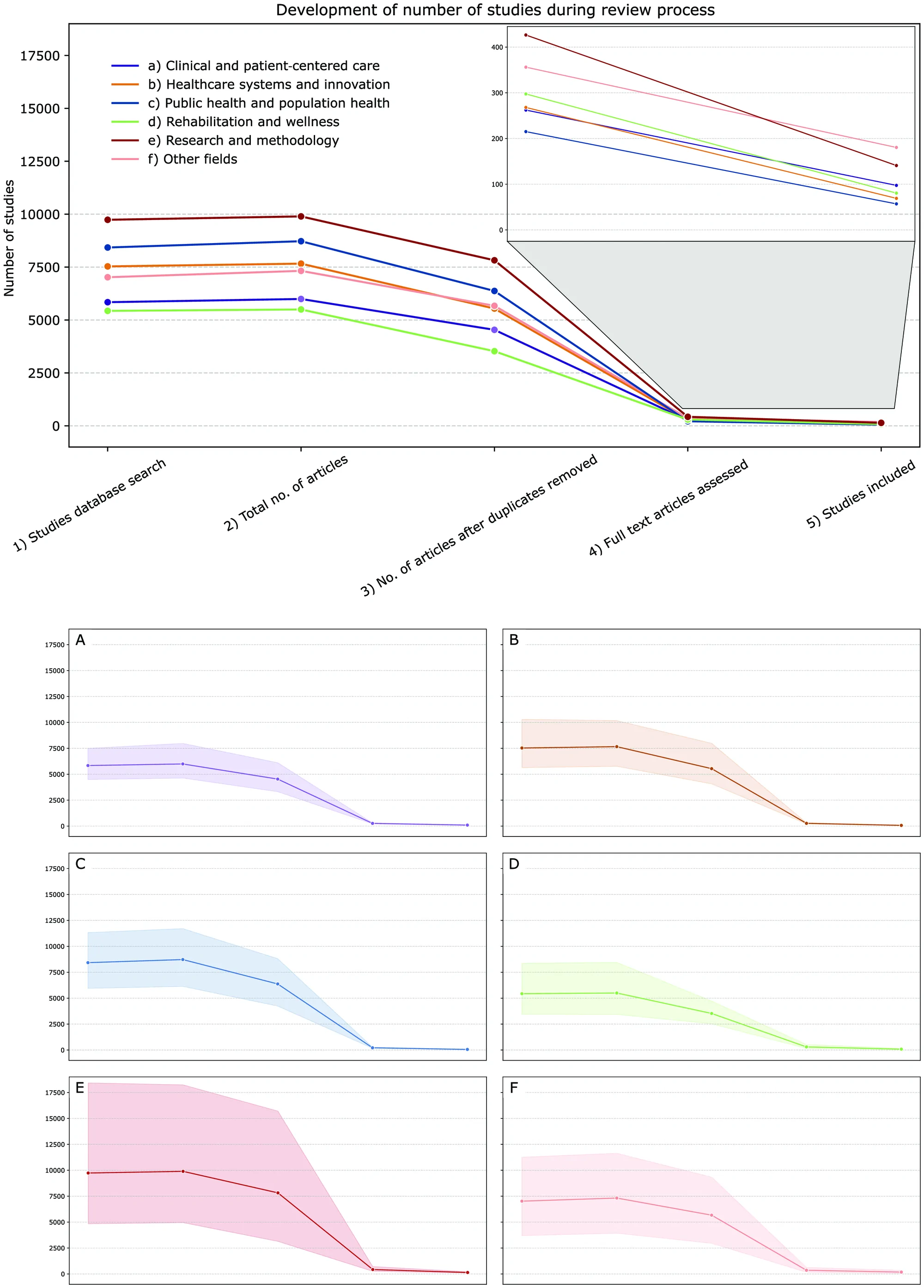
The number of studies at each stage of the review process is shown, starting from the database search, followed by the total number of publications (number of articles identified through database search and other sources combined), the number of publications after deduplication, full-text publications assessed, and studies ultimately included. The data are categorized by thematic group (a: Clinical and patient-centered care, b: Healthcare systems and innovation, c: Public health and population health, d: Rehabilitation and wellness, e: Research and methodology, f: Other fields), showing on a linear scale the average (solid line), as well as the minimum and maximum number of studies at each stage of the review process. Figure 4 long description.

A PRISMA diagram was missing in 27 publications (3.03%), while the remaining 864 publications provided data on search efficiency. On average, 6,920.66 studies (SD = 17,020.91; range: 54–289,844) were retrieved from databases and other sources, with 92.65 studies (SD = 252.74, range: 1–4,247) ultimately included per scoping review (see Figure 4), resulting in an average yield rate of 5.68% (SD = 0.10). Publications on research and methodology retrieved the highest number of studies during initial searches (10,005.26 studies, SD = 28,658.66), while those in the category “other fields” retrieved the fewest (7,322.42 studies, SD = 11,856.79). The widest range of study retrievals was observed in research and methodology (186–219,897 studies), also with the highest yield rate (8.11%). In contrast, publications covering healthcare systems and innovation had the lowest yield rate at 4.35%. In three articles on the topic of clinical and patient-centered care, AI tools (Continuous Active Learning tool, DistillerSR, or Research Screener) supported the screening process, prioritizing relevant articles and allowing researchers to discontinue screening once a predefined criterion was met.^24^^–^^26^ Initial searches identified an average of 116,579.67 studies across these publications, with yield rates ranging from 0.18% to 0.45%. Given these disproportionally low values, yield rates (overall and subgroup) were recalculated, excluding these AI-assisted studies. The overall yield rate increased from 5.68% to 5.70%, whereas the subgroup yield rate for clinical and patient-centered care increased from 6.17% to 6.21%.

The interrater reliability (e.g., kappa) was omitted in 758 publications (83.85%). When mentioned, these metrics were presented inconsistently, with some reporting them for full-text screening, some for abstract screening, and some for both.

Across 891 publications, 43 distinct screening tools were used, most frequently: EndNote (*n* = 248, 27.83%); Covidence (*n* = 226, 25.36%); Microsoft Excel (*n* = 201, 22.56%); Rayyan (*n* = 135, 15.15%); Mendeley (*n* = 51, 5.72%); Zotero (*n* = 22, 2.47%); Distiller SR (*n* = 18, 2.02%); Microsoft Word (*n* = 11, 1.23%); and Citavi (*n* = 10, 1.12%). Notably, 207 publications (23.23%) did not specify using any digital screening tool.

### 3.5 Framework use, language restriction, and appraisal practices

Most publications (*n* = 687, 77.10%) did not describe a formal framework to guide eligibility criteria. Among the remaining 204 publications that did, 141 (69.12%) used the PCC framework, 31 (15.20%) the Population, Intervention, Comparator, Outcome framework, and 22 (10.78%) the PICOS (S: Study design) framework. English language restrictions were reported in 335 publications (37.60%) during the study selection process, primarily excluding non-English studies (*n* = 255, 75.22%). Among the 13 non-English languages included alongside English, the most frequently represented were Spanish (*n* = 30, 3.37%); Portuguese (*n* = 30, 3.37%); French (*n* = 27, 3.03%); and German (*n* = 19, 2.13%).

Approximately, one-quarter of all publications (*n* = 219, 24.58%) reported conducting a critical appraisal, although 29.26% (*n* = 67) of publications did not specify the tool used. Some 243 publications (27.27%) explicitly stated that they did not conduct a critical appraisal, and 429 publications (48.15%) did not report on a critical appraisal. In total, 62 distinct critical appraisal assessment tools were used, the most common being the JBI appraisal tool (*n* = 34, 14.85%) and the Mixed Methods Appraisal Tool (*n* = 22, 9.61%). No rapid scoping review was conducted using critical appraisal, compared to 24.66% (*n* = 201) scoping reviews and 27.42% (*n* = 17) systematic scoping reviews. The likelihood of conducting a critical appraisal varied by screening tool: When Distiller was used for screening, 50.0% (*n* = 9) conducted a critical appraisal, while publications that used Citavi as a screening tool only conducted a critical appraisal in 10.0% (*n* = 1) of cases.

### 3.6 Funding

Of the 891 OSF registrations resulting in publications, 76.43% (*n* = 681) did not provide any information about funding, 11.56% (*n* = 103) explicitly reported no funding, and 12.01% (*n* = 107) declared funding. In contrast, among the associated 891 publications, 15.38% (*n* = 137) lacked funding information, 29.41% (*n* = 262) explicitly stated that no funding was received, and 55.22% (*n* = 492) acknowledged funding. Of these 492 publications, 29.47% (*n* = 145) provided further details about their funding sources: 40 % reported multisource funding (*n* = 58) and 31.03% government or state funding (*n* = 45). Funding was more frequent in high-income countries (*n* = 410, 57.42%) than in upper-middle-income countries (*n* = 69, 45.70%), lower-middle-income countries (*n* = 12, 52.17%), and low-income countries (*n* = 1, 33.33%). Funding did not show a correlation with the time to publish, number of authors, sector of the scoping review, number of studies included in a publication, or the presence of critical appraisal.

## 4 Discussion

Among the 891 publications analyzed, the vast majority were registered as “scoping reviews” (94.84%) on OSF, with 91.47% ultimately published as such. On average, 6.11 authors contributed to each review, which took 81.01 weeks from registration to publication for scoping and systematic scoping reviews, while rapid reviews were published within 55.63 weeks. Most scoping reviews (80.13%) originated from high-income countries, where they were more likely to receive funding. These scoping reviews mainly aimed to map evidence (44.78%) and assess interventions (28.40%). On average, searches were conducted across 5.21 databases, identifying 6,920.66 potential inclusions, with EndNote being the most commonly used screening tool. The average yield rate was 5.68%, including an average of 92.65 studies per review.

Regarding methodological rigor, 22.90% of reviews employed frameworks such as PCC or PICO to structure eligibility criteria. While PCC is widely recommended for scoping reviews due to its suitability for broad research questions, the use of PICO is generally less suitable.^11^ Language restrictions were applied to 37.60% of all reviews, and three-quarters of publications did not conduct a critical appraisal of the studies they included.

The dominance of high-income countries (80.13%) in our study aligns with previous findings, which reported 53% of scoping reviews as originating from North America and 38% from Europe.^27^ Additionally, our study found that 62.88% of all publications came from English-speaking countries, mirroring patterns observed in systematic reviews.^28^ Furthermore, our results confirmed that scoping reviews from high-income countries were more likely to have received funding, reinforcing previous research indicating that funding remains concentrated in high-income countries.^27^^,^^29^ This pattern is consistent across other research fields in healthcare, where low-income countries receive a fraction of the global funding and resources.^30^ Such disparities may introduce biases in research priorities and perspectives, potentially limiting the generalizability of findings to other geographic regions.^29^^,^^31^

Scoping reviews have become an important research methodology for evidence synthesis.^3^^,^^32^^,^^33^ In line with other research, our findings demonstrate a steady rise in scoping review registrations and publications from 2016 to 2023 across various disciplines, mirroring the increasing prevalence of systematic reviews over recent years.^32^^,^^34^ Our results indicate a 51.76% publication rate of protocols registered between 2016 and 2022, which was reported at 61.46% for systematic reviews.^28^ Although preregistration is optional, we observed an increase in registrations over the years. Studies suggest that only 21%–38% of systematic reviews include a protocol registration, with even lower rates for scoping reviews, ranging from 8.7 to 13%.^27^^,^^32^^,^^35^^,^^36^ Evidence comparing preregistered and unregistered scoping reviews is lacking. However, studies on systematic reviews show that prospective registration is associated with higher R-AMSTAR and PRISMA scores, indicating improved methodological quality and reporting.^37^ This underscores the need for further research on protocol registration and methodological practices. With more journals recommending registered protocols to publish reviews, protocol registrations might increase.^38^

Most scoping reviews—similar to systematic reviews—were conducted within the healthcare sector.^39^ Our analysis aligns with other studies, showing that nearly half of all scoping reviews aimed to map existing evidence, reflecting the core purpose of scoping reviews.^1^^,^^27^^,^^32^ Consequently, medical and health databases were the most frequently used for identifying relevant studies. Similar to systematic reviews in healthcare, MEDLINE is considered one of the most important databases.^40^ Our thematic subgroup analysis revealed differences in yield rate: Reviews focused on research and methodology retrieved the highest number of studies and achieved the highest inclusion rate (8.11%), whereas reviews addressing healthcare systems and innovation showed the lowest yield rate (4.35%). Prior research supports that systematic scope and search specificity affect the volume and relevance of retrieved studies.^41^^,^^42^

Although scoping reviews are relatively new, they have evolved to include different subtypes. Our study identified that 7.07% of reviews changed the labeling of the review type between registration and publication, with most transitioning from scoping reviews to systematic scoping reviews. During the COVID-19 pandemic, there was a notable rise in public health-related scoping reviews, reflecting the need for rapid research synthesis to guide virus transmission prevention and treatment development.^43^ Rapid scoping reviews, designed to accelerate the review process, were particularly common during this period to synthesize the most recent developments in outbreak control.^25^^,^^43^^–^^45^ In our study, rapid scoping reviews required 31% less time to be published than traditional scoping reviews, aligning with previous findings that rapid reviews take approximately 3.2 months compared to systematic reviews with an average of 14 months.^46^^,^^47^ While their speed is beneficial in urgent situations, rapid reviews may compromise comprehensiveness and methodological rigor.^48^ Our study found that systematic scoping reviews were more likely to conduct critical appraisal, suggesting an inclination toward the methodological rigor of systematic reviews.^49^

Only 24.58% of scoping reviews in our study conducted any form of critical appraisal. While current guidelines do not mandate critical appraisal assessment for scoping reviews, contrary to systematic reviews, this could be reconsidered, given their increasing role in informing policy and practice.^50^^,^^51^ However, since scoping reviews commonly include nonresearch articles such as policy documents, critical appraisal tools need to be carefully chosen to accommodate this type of information.

Similarly, while systematic reviews typically follow a standardized framework for eligibility criteria, only 22.90% (*n* = 204) of the scoping reviews in our sample used such a framework.^52^^–^^54^ This suggests that rigid frameworks may not always align with scoping review objectives. However, consistent with recommendations, all reviews in our sample included at least two authors.^55^ Our results of an average of 6.11 authors per publication align with systematic reviews (6.05 authors) and meta-analyses (6.77 authors).^56^

Language restrictions were reported in over one-third of reviews, potentially introducing bias and limiting generalizability, while in the remaining reviews, such restrictions may either not have been applied or not explicitly reported. Despite recommendations by Cochrane and the Campbell Collaboration, 49–84% of Cochrane and Systematic reviews apply language restrictions.^53^^,^^57^^,^^58^ Although most scientific literature is published in English, the inclusion of non-English literature can alter the outcome of the analysis.^59^^,^^60^ However, this presents additional challenges for researchers, including increased time.^61^ With the advancements of AI-based translation tools, integrating non-English literature may become more feasible in the future.^62^^,^^63^

Conducting systematic reviews and meta-analyses is time-consuming and can take up to 2,000 person-hours.^64^^,^^65^ An estimated 67.3 weeks to complete a systematic review further underscores the extensive time commitment.^12^ AI tools might greatly enhance efficiency in (large-scale) reviews, reducing manual effort in screening irrelevant records and saving up to 77% of the time taken to screen titles and abstracts.^66^^,^^67^ EndNote was the most frequently used screening tool in our study despite the availability of specialized AI tools such as Rayyan and Covidence, which suggests an awareness, accessibility, or acceptability gap.^68^ Additionally, our findings suggest that such screening tools might improve critical appraisal practices by enhancing consistency, efficiency, and reproducibility.^66^^,^^69^^,^^70^

### 4.1 Implications

Our findings provide valuable insights that can support researchers in planning and allocating time and resources for their scoping reviews. Additionally, these results can inform guideline updates, promoting greater methodological rigor and consistency in scoping review practices. This is particularly relevant given the underutilization of frameworks such as PCC and the widespread lack of a critical appraisal in scoping reviews. Enhancing critical appraisal assessment practices may also increase the adoption of literature screening tools, thus potentially improving efficiency and transparency in the screening process.

### 4.2 Limitations

While our study provides significant insights into scoping review metrics and methodology, it has several limitations. First, the analysis was restricted to protocols registered on the OSF. While OSF is an important protocol registration platform, it is not the only one, and including registrations from other platforms, such as Figshare, could provide a more comprehensive overview. Our findings should additionally be interpreted with caution when generalizing to nonregistered scoping reviews. Future studies should examine differences between registered and nonregistered scoping reviews. Second, some publications may have changed titles between protocol registration and publication, potentially leading to missed matches. To mitigate this, we searched Google, ResearchGate, and ORCID to identify authors and publications. Third, the estimated study duration in our analysis is constrained by the absence of data on the total person-hours required and the exact start date of work. Thus, the actual time from protocol registration to publication may differ from our calculated durations and does not indicate the actual workload. Finally, not all relevant methodological details were explicitly reported in publications. Despite careful data extraction efforts, this might affect the comprehensiveness of certain analyses.

## 5 Conclusion

This comprehensive study analyzed key metrics from 891 scoping reviews preregistered on OSF and published later, highlighting variety in framework application, language restrictions, and critical appraisal practices. The findings underscore areas for improvement in reporting quality and reduction of systematic bias in scoping reviews. Our results further provide guidance for researchers designing and conducting scoping reviews. Future research should prioritize integrating detailed methodological recommendations into scoping review guidelines and exploring the potential of automation technologies in streamlining research synthesis processes.

## Supporting information

10.1017/rsm.2026.10078.sm001 Nordmann et al. supplementary material Nordmann et al. supplementary material

## Data Availability

The data that support the findings of this study are available on request from the corresponding author, K.N.

## Supplementary material

To view supplementary material for this article, please visit http://doi.org/10.1017/rsm.2026.10078.

## Figure 1. Long description

On the left, three colored boxes in a horizontal sequence represent protocol identification and data extraction. The first box, labeled Protocol search and retrieval, details the database as Open Science Framework, search term as Scoping review, and protocol registration years as 2016 to 2023. The second box, Publication matching, lists search strategy as PubMed, Google, ResearchGate, O R C I D, and contacting first authors if full text was not accessible. The third box, Data extraction and coding, specifies data collected as review type, team size, duration, search characteristics, and other variables via Google Forms. From these steps, a vertical bar labeled Protocol registration n equals 2,038 anchors the Sankey diagram. Flow lines branch rightward into six outcome categories: No linked publication found n equals 990, Included for quantitative and qualitative analysis n equals 891 (highlighted with a green border), Ineligible n equals 125 with the note not published as a scoping review, Protocol registered after publication n equals 18, Full text not accessible n equals 11, and Duplicate protocols n equals 3. The width of each flow line visually represents the number of protocols in each category.

Navigate back to Figure 1..

## Table 1. Long description

From the top row, the left column is labeled Inclusion criteria and the right column is labeled Exclusion criteria. The first row lists All types of scoping reviews, including systematic scoping review and rapid scoping review, as inclusion, and Any other registration type as exclusion. The second row lists Full registrations as inclusion and Incomplete registrations or registration components as exclusion. The next three rows have only exclusion criteria: Registration withdrawn, Publication of the results within four weeks of registering the protocol, and Registration of the protocol after publication of results. The left column is blank for these rows.

Navigate back to Table 1..

## Table 2. Long description

From the top row, the table lists years from 2016 to 2023. For each year, the columns display: total number of registrations, total number of published articles by time of analysis, percentage of articles published by time of analysis, and average weeks to publish. In 2016, 5 registrations and 5 publications yield 100 percent published, with average weeks to publish marked as N slash A. In 2017, 5 registrations, 4 publications, 80 percent published, and 20.14 average weeks. In 2018, 43 registrations, 28 publications, 65.12 percent published, and 54.36 weeks. In 2019, 190 registrations, 106 publications, 55.79 percent published, and 63.10 weeks. In 2020, 846 registrations, 490 publications, 57.92 percent published, and 46.74 weeks. In 2021, 221 registrations, 103 publications, 46.61 percent published, and 61.75 weeks. In 2022, 313 registrations, 104 publications, 33.11 percent published, and 89.68 weeks. In 2023, 415 registrations, 51 publications, 12.29 percent published, and 99.76 weeks. The table shows a sharp increase in registrations in 2020, followed by a decline, while publication percentages and average weeks to publish fluctuate across years.

Navigate back to Table 2..

## Figure 2. Long description

The plot displays publication year on the x-axis from 2017 to 2024 and category on the y-axis, listing clinical and patient-centered care, healthcare systems and innovation, public health and population health, rehabilitation and wellness, research and methodology, and other fields. Each dot represents a publication, colored by subcategory as indicated in the legend on the right. The densest cluster is in clinical and patient-centered care, especially after 2020. Healthcare systems and innovation, and public health and population health also show increased activity post-2020. Subcategories are color-coded: blue for clinical and physical assessment, Covid-19, dental health, patient care and outcomes, psychology and behavioural studies, reproductive health, treatment and procedures; red for decision-making and communication, digital health and telemedicine, health workforce and training, healthcare delivery and systems; green for environment and occupation, health economics, public health and health policy, specialized care and public health, fitness and training; brown for rehabilitation and injury management, applied and translational research; purple for methodological frameworks, research ethics and dissemination, research improvement, study types, theoretical approaches; pink for animal studies and pharmacology. The legend lists all subcategories with corresponding colors. Publication density increases notably after 2020 across most categories.

Navigate back to Figure 2..

## Table 3. Long description

Starting from the top, the table presents three columns: category, total number of publications, and percent of publications. The first row shows 891 total publications, 100.00 percent. Under ‘Type of scoping review published’, scoping review: 815, 91.47 percent; systematic scoping review: 62, 6.96 percent; rapid scoping review: 7, 0.79 percent; others: 7, 0.79 percent. Under ‘Country G N I classification’, high: 714, 80.13 percent; upper-middle: 151, 16.95 percent; lower-middle: 23, 2.58 percent; low: 3, 0.34 percent. Under ‘Databases’, medicine and health: 857, 96.18 percent; multidisciplinary: 516, 57.91 percent; engineering and technology: 377, 42.31 percent; systematic reviews databases: 295, 33.11 percent; social sciences and humanities: 67, 7.52 percent; open access repositories: 37, 4.15 percent; government and policy: 9, 1.01 percent; gray literature and websites: 8, 0.90 percent; theses and dissertations: 7, 0.79 percent; other specialized databases: 125, 14.03 percent. Under ‘Aims’, map existing evidence: 399, 44.78 percent; assess interventions: 253, 28.40 percent; identify gaps: 111, 12.46 percent; understand outcomes and impact: 87, 9.76 percent; develop new frameworks and models: 66, 7.41 percent; identify barriers and facilitators: 21, 2.36 percent. Under ‘Eligibility criteria’, P C C: 141, 15.82 percent; P I C O: 31, 3.48 percent; P I C O S: 22, 2.47 percent; other frameworks: 10, 1.12 percent; no framework reported: 687, 77.10 percent. Under ‘Eligible primary sources of evidence’, research articles: 881, 98.88 percent; gray literature: 313, 35.13 percent; abstracts: 43, 4.83 percent; books and chapters: 22, 2.47 percent; letters and commentaries: 15, 1.68 percent; editorials: 10, 1.12 percent; organizational reports: 6, 0.67 percent; other sources: 15, 1.68 percent. Under ‘Critical appraisal’, yes: 219, 24.58 percent; no: 243, 27.27 percent; not available: 429, 48.15 percent. Under ‘Screening tools’, one or more screening tools: 684, 76.77 percent; none or none reported: 207, 23.23 percent. Under ‘Funding’, yes: 492, 55.22 percent; no funding received: 262, 29.41 percent; no funding reported: 137, 15.38 percent.

Navigate back to Table 3..

## Figure 3. Long description

This choropleth map uses shades of blue to represent the number of publications per country, with a gradient scale at the top center labeled ‘No. of publications’ ranging from 1 on the left to 164 on the right. The darkest blue regions are the United States, Canada, Australia, and several countries in Western Europe, indicating the highest publication counts. Moderate blue shades appear in Brazil, China, Japan, and India. Most African, Middle Eastern, and Southeast Asian countries are shaded very light blue, indicating lower publication counts. Countries with no data or zero publications are shown in gray. The spatial distribution reveals a concentration of publications in North America, Western Europe, and Australia, with lower counts in Africa, Central Asia, and parts of South America.

Navigate back to Figure 3..

## Figure 4. Long description

The main panel displays a line graph with the x-axis labeled from left to right as Studies database search, Total number of articles, Number of articles after duplicates removed, Full text articles assessed, and Studies included. The y-axis shows Number of studies from 0 to 17500. Six colored lines represent thematic groups: a) Clinical and patient-centered care (blue), b) Healthcare systems and innovation (red), c) Public health and population health (teal), d) Rehabilitation and wellness (green), e) Research and methodology (pink), f) Other fields (brown). All groups show a sharp decline in study numbers at each stage, with the steepest drop after deduplication. Solid lines indicate averages, shaded areas show minimum and maximum values. An inset at the top right zooms in on the final stages, highlighting the small numbers included. Below, six panels labeled A to F correspond to each group, arranged left to right, top to bottom. Each panel shows the same x and y axes, with a colored line and shaded area for that group. All panels display a consistent pattern: high initial counts, a marked drop after deduplication, and very few studies included at the end.

Navigate back to Figure 4..
