# Supplementary material for: Meta-research on key metrics of preregistered scoping reviews
Source: Res Synth Methods. 2026 Jun 15;17(6):1101–17. doi: 10.1017/rsm.2026.10078 (PMC13732362; doi:10.1017/rsm.2026.10078)
Supplement: Supplementary file 1 [file S1759287926100787sup001.zip › S1759287926100787sup001/Appendix_2_Google_Form_V2_Ausformuliert.docx]

**Formularbeginn**

**Scoping Reviews**

**Registration (page 1)**

1. In which **database** was the scoping review registered?

*Select only one option.*

⬭ OSF

⬭ JBI

2. Which **type of scoping review** was registered?

*Select only one option.*

⬭ Scoping review

⬭ Rapid scoping review

⬭ Systematic scoping review

⬭ Other: _________________

3. Provide the **link to the registration**.

__________________________________________________________________

4. Provide the **date of the first registration**. *(example 07. January 2019)*

__________________________________________________________________

5. How many **team members** were named in the registration?

__________________________________________________________________

6. Was **funding** mentioned in the scoping review registration?

*Select only one option.*

⬭ Yes, funding was mentioned

⬭ No, no funding was mentioned

⬭ No information regarding funding was provided in the registration

7. If in the registration no data was provided about the scoping review or the registration was withdrawn, the registration will be **excluded from the analysis**. In case this applies, select one option below.

*Select only one option.*

⬭ Exclude: no information provided

⬭ Exclude: withdrawn

8. Were the **results** of the scoping review **published** in the form of a **scholarly article**?

*Select only one option.*

⬭ Yes *(Proceed with question 12)*

⬭ No *(terminate the survey after page 1)*

9. If the results were **published in another way than a scholarly article**, please specify and – if possible – provide the link:

_________________________________________________________________

10. If the **scoping review protocol was published in form of a scholarly article**, include the link to the protocol here.

_________________________________________________________________

11. Any other **observations**?

_________________________________________________________________

**Results (page 2)**

12. Provide the **link to the published article**.

__________________________________________________________________

13. If available, provide the **date of the manuscript was received** *(example 07. January 2019)*

__________________________________________________________________

14. If available, provide the **date the manuscript was revised** *(example 07. January 2019)*

__________________________________________________________________

15. If available, provide the **date the manuscript was accepted** *(example 07. January 2019)*

__________________________________________________________________

16. If available, provide the **date the manuscript was published** *(example 07. January 2019)*

__________________________________________________________________

17. How many people **authored** the publication?

__________________________________________________________________

18. How may **authors of the publication were team members** in the registration?

__________________________________________________________________

19. Indicate the country of first author's institution. If there are multiple affiliations, choose the country of the first mentioned affiliation.

*Select only one option.*

⬭ Australia

⬭ Brazil

⬭ Canada

⬭ China

⬭ France

⬭ Germany

⬭ India

⬭ Indonesia

⬭ Iran

⬭ Italy

⬭ Japan

⬭ Netherlands

⬭ Poland

⬭ Russia

⬭ South Korea

⬭ Spain

⬭ Switzerland

⬭ Turkey

⬭ The United Kingdom

⬭ The United States

⬭ Other: _________________

20. Which **type of scoping review** was reported in the article?

*Select only one oval.*

⬭ Scoping review

⬭ Rapid scoping review

⬭ Systematic scoping review

⬭ Other: _________________

21. Copy the **abstract** here.

__________________________________________________________________

__________________________________________________________________

__________________________________________________________________

__________________________________________________________________

22. Copy the **aim and/or research questions** here.

__________________________________________________________________

__________________________________________________________________

__________________________________________________________________

__________________________________________________________________

23. How many **databases** were searched?

__________________________________________________________________

24. Indicate the **databases** that were used.

*Select all applicable answers.*

☐ AMED

☐ BioMed Central Databases

☐ CINAHL

☐ Cochrane Library

☐ EBSCO

☐ Embase

☐ Google Scholar

☐ LILACS

☐ MEDLINE

☐ PubMed

☐ ProQuest

☐ PsycINFO

☐ ScienceDirect

☐ Scopus

☐ Web of Science

☐ Other: _________________

25. Indicate all **resources that were included for analysis**.

*Select all applicable answers.*

☐ Articles

☐ Books/chapters

☐ Conference abstracts

☐ Editorials

☐ Grey literature

☐ Other: _________________

26. Copy the **inclusion criteria** here.

__________________________________________________________________

__________________________________________________________________

__________________________________________________________________

__________________________________________________________________

27. Copy the **exclusion criteria** here.

__________________________________________________________________

__________________________________________________________________

__________________________________________________________________

__________________________________________________________________

28. Copy the **search strategy** here.

__________________________________________________________________

__________________________________________________________________

__________________________________________________________________

__________________________________________________________________

29. Which **tool(s) were used for screening**?

*Select all applicable answers.*

☐ Citavi

☐ Covidence

☐ DistillerSR

☐ Endnote

☐ Microsoft Excel

☐ Microsoft Word

☐ Rayyan

☐ RevMan

☐ NONE / NONE reported

☐ Other: _________________

30. Was a **PRISMA diagram** provided?

*Select only one option.*

⬭ Yes

⬭ No

31. Indicate the **number of studies found through database search**.

__________________________________________________________________

32. Indicate the **number of additional records found through other sources.**

__________________________________________________________________

33. Indicate the **number of duplicates removed.**

__________________________________________________________________

34. Indicate the **number of records excluded in the abstract screening phase.**

__________________________________________________________________

35. Indicate the **number of full text articles assessed for eligibility.**

__________________________________________________________________

36. Indicate the **number of excluded after full text screening.**

__________________________________________________________________

37. Indicate the **number of studies included in the scoping review.**

__________________________________________________________________

38. If necessary, provide **comments on the PRISMA flow diagram** and/or the numbers provided in question(s) 32-37.

__________________________________________________________________

39. If the **interrater agreement** was mentioned, include it here.

__________________________________________________________________

40. Was any information about **funding** mentioned in the article?

*Select only one option. Choose yes only if funding for the whole review was reported. If general statements about salary support were mentioned or conflict of interests of specific authors disclosed, choose “no, no funding reported”.*

⬭ Yes

⬭ No, no funding reported

⬭ No, no information available

41. If there are **explicit statements of author salary support**, please include them here.

__________________________________________________________________

42. If available, provide a **link to all studies included in the scoping review**.

__________________________________________________________________

43. For internal management of articles, please save the article as a PDF using the following format: SurnamefirstauthorYear and copy the **name of the PDF** here.

__________________________________________________________________

44. If necessary, include here **other observations** about the article?

__________________________________________________________________

Formularende
